# Towards the Use of Standardized Terms in Clinical Case Studies for Process Mining in Healthcare †

**DOI:** 10.3390/ijerph17041348

**Published:** 2020-02-19

**Authors:** Emmanuel Helm, Anna M. Lin, David Baumgartner, Alvin C. Lin, Josef Küng

**Affiliations:** 1 Research Department Advanced Information Systems and Technology, University of Applied Sciences Upper Austria, 4232 Hagenberg, Austria; anna.lin@fh-hagenberg.at (A.M.L.); david.baumgartner@fh-hagenberg.at (D.B.); 2 Institute for Applied Knowledge Processing, Johannes Kepler University, 4040 Linz, Austria; josef.kueng@jku.at; 3 Faculty of Medicine, University of Toronto, Toronto, ON M5S 1A8, Canada

**Keywords:** process mining, healthcare, terminology, ICD, SNOMED

## Abstract

Process mining can provide greater insight into medical treatment processes and organizational processes in healthcare. To enhance comparability between processes, the quality of the labelled-data is essential. A literature review of the clinical case studies by Rojas et al. in 2016 identified several common aspects for comparison, which include methodologies, algorithms or techniques, medical fields, and healthcare specialty. However, clinical aspects are not reported in a uniform way and do not follow a standard clinical coding scheme. Further, technical aspects such as details of the event log data are not always described. In this paper, we identified 38 clinically-relevant case studies of process mining in healthcare published from 2016 to 2018 that described the tools, algorithms and techniques utilized, and details on the event log data. We then correlated the clinical aspects of patient encounter environment, clinical specialty and medical diagnoses using the standard clinical coding schemes SNOMED CT and ICD-10. The potential outcomes of adopting a standard approach for describing event log data and classifying medical terminology using standard clinical coding schemes are further discussed. A checklist template for the reporting of case studies is provided in the Appendix A to the article.

## 1. Introduction

Process mining is a discipline that allows for greater understanding into real-life processes of recorded systems behaviour. Through process mining techniques, numerous case studies and successful companies have demonstrated further quality improvement, compliance, and optimization of processes.

In healthcare, recent review papers have provided an overview of process mining across clinical case studies. Rojas et al in 2016 identified eleven common aspects across 74 clinical case studies [1]. These aspects include methodologies, techniques or algorithms, medical fields and healthcare specialty. In 2018, Erdogan and Tarhan conducted a systematic mapping of 172 case studies with mostly the same metrics and aspects [2]. These papers are very specific as to *how* these case studies were conducted, which enhances comparison between different process mining techniques in different settings. However, from a medical perspective, the terms and categories listed under *medical fields* and *healthcare specialty* are not structured in a uniform way, and do not follow a standardized clinical coding scheme. Further, basic characteristics of the event log data (timeframe, number of cases or patients, healthcare facility/organization) are not always clearly reported.

The number of case studies on process mining in healthcare continues to increase steadily. As such, a standard approach of reporting event log data, clinical specialties and medical diagnoses would provide greater clarity and enhance comparability between treatments of specific diseases across different heathcare settings.

In this article, further to the studies examined by Rojas et al., we conducted a forward search of processing mining case studies in healthcare for the three-year period from January 2016 to December 2018. We identified case studies that described basic characteristics of the event log data, and where information on the patient encounter environment, clinical specialty and medical diagnoses could be assigned under a standard clinical coding scheme. Section 2 describes how the forward search was conducted and which criteria we applied to filter the results. In addition, the methods describe standard clinical coding systems and terminologies that were used. In Section 3, the results of our analysis are presented. Section 4 discusses the benefits and gives an outlook on the potential clinical insights gained by reporting and classifying clinical terms, clinical specialties and medical diagnoses using a standard clinical coding scheme.

This article is an extension to the paper presented at the workshop on Process-Oriented Data Science for Healthcare 2019 (PODS4H19), held in conjunction with the BPM2019 conference in Vienna, Austria [3]. It presents further details to the results of our analysis and it provides an outline for a reporting template in Appendix A. This template can be used as a checklist for the reporting of case studies on process mining in healthcare.

## 2. Materials and Methods

Our paper focused on answering three questions: (1) Which clinically-relevant case studies of process mining in healthcare will be selected for this study? (2) What were the technical aspects identified? (3) How can we improve the clarity and comparability of the clinical terms and aspects described?

### 2.1. Selection of Clinically-Relevant Case Studies

**Figure 1:**
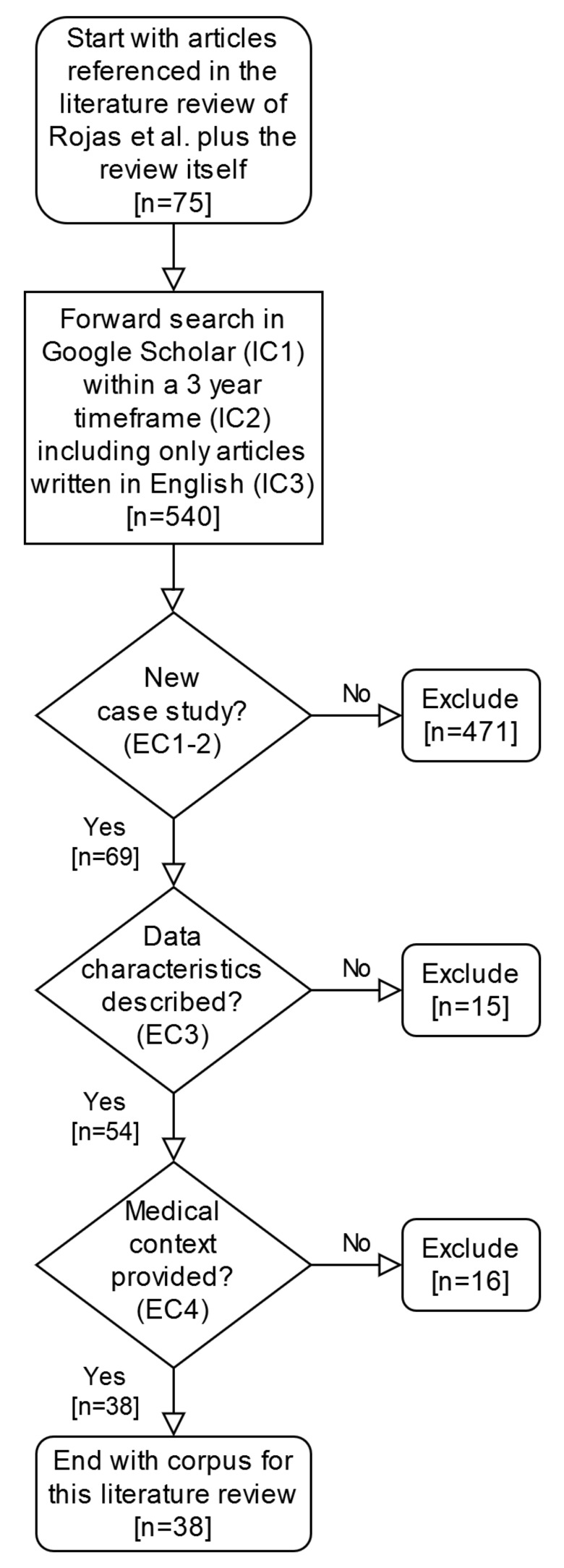
Flowchart on the case study selection strategy.

Our starting point was the review paper by Rojas et al. [1] which identified 74 case studies where process mining tools, techniques or algorithms were applied in the healthcare domain. We then performed a forward search using Google Scholar, in reference to the 74 identified articles and the review paper itself. The inclusion criteria (IC) were applied at once in the Google Scholar search and the exclusion criteria (EC) were applied manually afterwards (see Figure 1).

- IC1: All articles that reference either the review paper by Rojas et al. [1] or any of the 74 articles identified in their review were included.
- IC2: All articles published between 01.01.2016 and 31.12.2018 were included.
- IC3: All articles published in English were included.

- EC1: Articles that do not include evidence of a clinically-relevant case study of process mining in healthcare were excluded.
- EC2: Articles that present a case study based on data that was already used for an earlier case study were excluded.
- EC3: Articles that do not describe the characteristics of the event log data (e.g., timeframe, number of cases or patients, healthcare facility) or do not describe which process mining technique or algorithm was applied were excluded.
- EC4: Articles that did not describe any clinical context (i.e., clinical specialty or medical diagnosis) were excluded.

### 2.2. Technical Aspects

A detailed account of the tools, techniques or algorithms used in process mining case studies in healthcare have been previously described [1]. Also, other technical descriptors such as the data type and geographical analysis have been used to describe the event log data [4]. For the technical scope of our paper, our focus was on (1) the *tools* used in the case studies, (2) the *techniques or algorithms* used, and (3) the *process mining perspectives*.

### 2.3. Clinical Aspects and Standard Coding Schemes

Medical language is full of homonyms, synonyms, eponyms, acronyms and abbreviations; and each healthcare specialty comes with their own sub-terminology [5]. To improve the clarity and comparability of the clinical aspects described in our selected papers, we adopted the use of standard clinical coding schemes of SNOMED CT and ICD-10. Namely, the clinical terms were matched to their best corresponding standard clinical descriptor, with respect to three clinical categories: (1) the type of patient encounter environment (2) clinical speciality and (3) medical diagnosis (i.e., disease or health problem).

#### 2.3.1. SNOMED CT

The Systematized Nomenclature of Medicine – Clinical Terms is an internationally recognized standard that classifies clinically-relevant terminology and concepts, along with their synonyms and relationships, into numeric coded values. Available in multiple languages and maintained by SNOMED International, there are currently over 340,000 numerically coded concepts that can be combined grammatically to create an expression. We used SNOMED CT international browser (https://browser.ihtsdotools.org/) in version *v20190131* for clinical descriptors on the *Patient encounter environment* and *Clinical specialty*.

#### 2.3.2. ICD-10

For classification of clinical diagnoses and health problems, the commonly accepted system is the International Classification of Diseases or ICD, which is maintained by the World Health Organization (WHO). The most current version is ICD-10 and it utilizes an alphanumeric coding scheme with more than 14.000 single clinical codes of medical terms organized hierarchically into 22 chapters. We used the WHO ICD-10 browser in the *2016* version (https://icd.who.int/browse10/2016/en) for clinical descriptors on *medical diagnoses*.

## 3. Results

### 3.1. Selection of Clinically-Relevant Case Studies

Our forward search yielded initially a total of 540 papers, and after our inclusion and exclusion criteria were applied, 38 articles were selected (cf. Figure 1). For all 38 papers, basic characteristics of the event log data were retrieved (e.g., origin of data, number of cases or patients, healthcare facility, timeframe of the study). The results of the technical and clinical aspects are described below.

### 3.2. Technical Aspects

#### 3.2.1. Tools

Table 1 summarizes our findings of the most commonly used Tools to enable process mining techniques and algorithms. ProM (https://www.promtools.org) was the most frequent, found in 18 of the selected case studies, and was also found of the same in [1]. Nowadays Disco (https://fluxicon.com/disco) is becoming more prevalent, and we found 11 cases as well. To complete our analysis, PALIA was used twice; and in both cases, this tool was used in combination with another tool or technique.

There are a wide variety of other Tools used, often together with ProM, which are listed together in the table for a total of 13 papers. Six case studies introduced self-developed tools.

#### 3.2.2. Techniques or Algorithms

Table 2 describes the four most used techniques and algorithms amongst the selected case studies. Our analysis revealed that *Fuzzy miner* (as implemented in Disco) was most frequently used, appearing in 11 of the case studies. Of note, several papers that utilized ProM also presented self-developed approaches that were case-specific based on the ProM environment. Further, the *Inductive visual miner* is one of the more recent built-in miners in ProM, and is now more frequently used and reported as such. Five case studies used the Trace Clustering technique. Other types like BPMN, ANOVA and machine learning were sometimes used but not on a frequent basis. While the Heuristic miner algorithm was frequented as per previous reviews, [1,2], it was only used in two of our 38 selected papers.

#### 3.2.3. Process Mining Perspectives

Our analysis showed that the majority of the case studies (30 in the total) mainly aimed for the *Control Flow* perspective in their dataset (see Table 3). Of those remaining, five papers analyzed the *Conformance* perspective, two focused on *Organizational*, and one on *Performance*.

### 3.3. Clinical Aspects using Standard Clinical Descriptors

#### 3.3.1. Encounter Environment

From the patient’s perspective, we considered five clinical settings or encounter environments: (1) Inpatient, (2) Outpatient, (3) Accident and Emergency department or AED, (4) General practitioner or GP practice site, and (5) Pharmacy. All five encounter environments could be coded by SNOMED CT. For each paper, at least one of these five encounter environments was retrieved. Most of the papers examined events within the Inpatient environment, followed by AED environment (cf. Table 4).

#### 3.3.2. Clinical Specialty

SNOMED CT offers the code of 394658006 for *Clinical specialty*, which further contains 18 high-level specialties. Table 5 shows 11 of the 18 high-level clinical specialties were identified in our selected papers. The most identified clinical specialty was *Medical specialty*, followed by *Surgical specialty* and *Emergency medicine*. Of note, some of 18 high-level specialties in SNOMED CT are further divided into sub-specialties of greater clinical specificity. For example, *Medical specialty* has 44 sub-specialties that include e.g., *Dermatology*, *Neurology* and *Cardiology*. In this paper, we identified and assigned sub-specialities to their corresponding high-level *Clinical specialty*. Also, for example, if several different medical sub-specialities were described in one paper, we counted these sub-specialities together as *Medical specialty*.

#### 3.3.3. Medical Diagnosis

For each paper, we focused on identifying the medical diagnosis (i.e., disease or health problem) or description of a medical diagnosis. We then assigned these terms to their corresponding highest chapter or block category in ICD-10. Table 6 shows a total of 15 out of the 22 ICD-10 chapter categories for disease and health related problems were covered amongst the papers. The category with the most papers listed was *Diseases of the circulatory system* followed by *Neoplasms*. Two papers [16,21] were not included in Table 6, since several hundred diseases and health problems were cited and classified using ICD-9. Of the remaining 36 case studies, ICD-10 was already used in 8 papers to code the diagnosis [12,14,21,22,33,34,38,40].

## 4. Discussion

Whether for process discovery, conformance checking, or enhancement, process mining case studies are influenced by the quality of the labeled data. The benefits of high-quality, labeled data include improved accuracy, efficiency and predictability of processes, not only for the study itself but also for comparability across studies. Further, high-quality, labeled data can make other kinds of future analyses and even machine learning techniques (e.g., supervised learning, trend estimation, clustering) easier and more efficient to achieve. In process mining case studies in healthcare, labeled data often encompasses clinical aspects and terms. As such, our aim was to examine clinically-relevant case studies since Rojas et al. [4] and determine how to improve upon the clarity and comparability of clinical aspects and terms described.

### 4.1. Reporting Basic Characteristics of the Event Log Data

For our analysis, we selected papers that described basic characteristics of the event log data. These characteristics included the origin or source of the data, the healthcare facility, the number of cases or patients, and the timeframe of the study. For example, in Rinner et al. [18], event logs were extracted for a total of 1023 patients starting melanoma surveillance between January 2010 to June 2017, from a local melanoma registry in a medical university and Hospital Information System (HIS) in Austria. In papers where these characteristics were not clearly reported, the retrieval process was time-consuming. Several papers provided additional details (e.g., patient age, data from private insurance or public health records). Presumably for reasons of privacy and anonymity, specifics on the healthcare facility (e.g., hospital name) were not always provided, however, the country of origin was always reported. While variations exist in the style of reporting, we recommend case studies include these aforementioned basic characteristics when reporting the event log data.

### 4.2. Adopting the Use of Standard Clinical Descriptors

#### 4.2.1. Encounter Environment

A patient can have vastly different experiences within the healthcare system depending on the clinical setting or encounter environment. For example, a patient with heart failure who presents to the AED may require admission as a hospital inpatient, follow-up at their GP practice site or outpatient clinic, and prescription drugs at a pharmacy. As such, in our analysis of the selected papers, we focused on five patient encounter environments: Inpatient, Outpatient, AED, GP practice site, and Pharmacy. All five encounter types can be coded by SNOMED CT. While further details can be provided (e.g., Outpatient Clinic for Thyroid Disease [32]), we recommend case studies report at least the patient encounter environment using standard clinical codes e.g., SNOMED CT.

#### 4.2.2. Clinical Specialty

Different clinical specialties are often involved in the care of a patient. For example, for a patient diagnosed with cancer, a multidisciplinary care plan can encompass input from a medical specialty, a surgical specialty and clinical oncology. As each specialty offers their own unique set of knowledge and expertise, it is important to identify which clinical specialty is involved.

For each of our selected papers, we identified at least one of the 18 high-level clinical specialties coded by SNOMED CT. For greater specificity, SNOMED CT offers further standard clinical codes for sub-specialities. In fact, Baek et al. list multiple sub-specialities along with their corresponding SNOMED CT codes in their study [12]. Also, instead of *Clinical specialty*, another category of clinical descriptors such as the type of medical practitioner or occupation could have been considered (e.g., mapping to surgeon instead of surgical specialty).

In any event, the task of identifying and assigning such standard clinical codes is time consuming, and beyond the scope of this paper. For future case studies, we recommend reporting the clinical specialty (or similar clinical descriptor such as medical practitioner) by adopting standard clinical codes e.g., SNOMED CT.

#### 4.2.3. Medical Diagnosis

There are literally thousands of medical diagnoses, and each diagnosis comes with its own treatment and management plan. ICD-10 is a standard coding scheme in healthcare that provides specific clinical descriptors and codes for diseases and health conditions. In our analysis, we were able to identify at least one medical diagnosis or description of a medical diagnosis in each paper, which we could map to the corresponding ICD-10 code. Further, over 25% (10 out of 38) of our selected papers utilized either ICD-9 or ICD-10 codes in their study. For broader comparison across studies, we assigned the selected papers to one or more of the 22 ICD-10 chapters or block categories. In Table 6 we only listed the ICD-10 chapters that were covered in the case studies.

It is important to distinguish the difference between a medical diagnosis (i.e., the process of identifying the disease or medical condition that explains a patient’s signs and symptoms) versus a patient’s signs (e.g., rash) or symptoms (e.g., cough). While the majority of ICD-10 chapters describe a group of medical diagnoses, some cover other clinical descriptors, such as signs and symptoms (R00-R99), external causes of morbidity and mortality (V01-V98), and codes for special purposes (U00-99). ICD-10 also allows for the coding of location, severity, cause, manifestation and type of health problem [43].

Taken together, we recommend adopting use of a standard coding scheme e.g., ICD-10 for clinical terms and aspects relating to medical diagnosis in process mining case studies in healthcare. Recently developed, ICD-11 is not adopted yet but provides backward compatibility, i.e., ICD-10 coded case studies will be comparable to newer ICD-11 coded ones, once the new coding scheme will be taken on by the information system vendors.

### 4.3. Conclusions and Future Perspectives

In summary, we propose adopting a standard for describing event log data and reporting medical terminology using standard clinical descriptors and coding schemes. In doing so, the goal is to improve accuracy and comparability across future clinically-relevant process mining case studies in health care. As such, we provide a sample checklist template of standard criteria for the reporting of such case studies, in Appendix A.

In scientific research, the idea of having a set of guidelines, criteria, or standards for peer-reviewed publications is not novel. In fact, journals such as *Nature* are taking initiatives by creating mandatory reporting summary templates (https://www.nature.com/documents/nr-reporting-summary-flat.pdf), in order to improve comparability, transparency, and reproducibility of the work they publish [44]. Other journals and disciplines, including biomedical informatics, are following suit [45]. Thus, as data sets become more transparent and available, consistency in reporting characteristics of the event log data (e.g., origin of data, number of patients or cases, healthcare facility, timeframe of the study) will aid in improving comparability and reproducibility across future studies.

Further to the work by Rojas et al. [28], we identified and described the clinical terms and aspects in our selected papers with respect to three categories: the patient encounter environment, clinical specialty, and medical diagnosis. We then correlated the clinical terms and aspects to their respective standard clinical descriptors and codes found in SNOMED CT and ICD-10. For studies where a higher granularity for patient encounter environments is needed, SNOMED CT offers more codes and the compositional grammar could be useful. Similarly, for *Clinical specialty* in SNOMED CT, reporting of sub-specialties under e.g., *Medical speciality* will provide increased specificity for clarity and comparison.

As aforementioned, several case studies have already adopted the use of a standard clinical coding scheme to describe medical diagnoses. Howevever, our consideration of SNOMED CT and ICD-10 serves only as a starting point. In fact, SNOMED CT also provides standard codes for medical diagnoses, which can provide further specificity and clarity. For example, instead of ICD-10, the one of Systematized Nomenclature for Dentistry or SNODENT CT (which is incorporated into SNOMED CT) could have been used to code for the clinical descriptors of missing and filled teeth in one of our selected papers [7].

Finally, when adopting the use of standard clinical descriptors, we recognize other fundamental clinical categories to consider are medical investigations and procedures. As such, the use of standard clinical descriptors is becoming increasingly relevant, not only for clarity and comparability, but efficiency in outcome measurements such as length of stay (LOS) and financial cost. For example, in their paper, Baek et al. utilized process mining techniques and statistical methods to identify the factors associated with LOS in a South Korean hospital [12]. This study is just one use case for a more detailed description of the medical context where process mining case studies could allow for future meta-studies, e.g., benchmarking LOS in different hospitals or countries, based on diagnoses while also considering other important factors like the patient encounter environment.

## Figures and Tables

**Table 1:** Papers with their corresponding Tools most commonly used (non-disjoint). In some papers, other tools were developed or used, resulting in two categories of *Others* and *Self-developed*.

Tool	ProM	Disco	PALIA	pMineR	Others	Self-Developed
**Papers**	[6,7,8,9,10,11,12,13,14,15,16,17,18,19,20,21,22,23]	[4,7,8,9,18,24,25,26,27,28,29]	[30,31]	[32]	[6,12,19,25,29,30,31,33,34,35,36,37,38]	[16,27,33,39,40,41]

**Table 2:** Papers with their corresponding Techniques or Algorithms that were mainly used.

Techniques/Algorithms	Fuzzy Miner	Self-Developed	Clustering	Heuristic Miner
**Papers**	[4,7,8,9,18,24,25,26,27,28,29]	[10,11,13,15,16,27,37,39,40,41,42]	[6,26,32,33,34]	[10,21]

**Table 3:** Papers with their corresponding process mining perspectives.

Perspectives	Control Flow	Conformance	Organizational	Performance
**Papers**	[4,6,7,10,11,12,13,14,15,16,17,19,20,22,23,25,26,27,28,29,30,33,34,35,36,37,38,39,41,42]	[9,18,21,32,40]	[24,31]	[8]

**Table 4:** Papers with their corresponding SNOMED CT encounter environment.

SNOMED CT	Environment	Papers
440654001	Inpatient	[4,6,8,11,12,13,14,15,16,18,19,20,21,22,23,25,26,27,28,29,33,34,35,36,37,38,39,40,41,42]
440655000	Outpatient	[7,9,17,32,38]
225728007	AED	[10,11,13,17,19,22,24,27,28,30,35,36]
394761003	GP practice site	[31]
264372000	Pharmacy	[4]

**Table 5:** Papers with their corresponding SNOMED CT clinical specialty.

SNOMED CT	Clinical Specialty	Papers
394592004	Clinical oncology	[14,37,38]
394581000	Community medicine	[31]
722163006	Dentistry	[7,9]
722164000	Dietetics and nutrition	[31]
773568002	Emergency medicine	[10,11,12,13,17,19,22,24,28,30,35,36]
394814009	General practice	[7,9,31]
408446006	Gynecological oncology	[41]
394733009	Medical specialty	[4,6,9,12,15,16,18,21,22,25,29,32,33,34,35,39,40,42]
722165004	Nursing	[9,24,31]
394585009	Obstetrics and gynecology	[16,41]
394732004	Surgical specialty	[8,9,11,12,14,20,23,26,32,37,41]

**Table 6:** Papers with their corresponding ICD-10 medical diagnosis.

ICD-10	Diagnosis	Papers
A00 - B99	Certain Infectious and parasitic diseases	[4,12,13,19,22,36]
C00 - D48	Neoplasms	[12,14,18,20,25,37,38,41]
E00 - E90	Endocrine, nutritional and metabolic diseases	[9,12,15,31,32,42]
F00 - F99	Mental and behavioural disorders	[12,40]
G00 - G99	Diseases of the nervous system	[12]
H60 - H95	Diseases of the ear and mastoid process	[12]
I00 - I99	Diseases of the circulatory system	[6,8,12,15,25,30,33,34,35,39,42]
J00 - J99	Diseases of the respiratory system	[12,24]
K00 - K93	Diseases of the digestive system	[7,12,24,28]
M00 - M99	Diseases of the musculoskeletal system and connective tissue	[12,24,40]
N00 - N99	Diseases of the genitourinary system	[12]
O00 - O99	Pregnancy, childbirth and the puerperium	[12]
R00 - R99	Symptoms, signs and abnormal clinical and laboratory findings, not elsewhere classified	[10,12,24]
S00 - T98	Injury, poisoning and certain other consequences of external causes	[10,11,12,17,24,27,29]
Z00 - Z99	Factors influencing health status and contact with health services	[12,23,26,32,37]

## Appendix A. Reporting Template Outline

The following sections can be used as a checklist template when writing a case study on process mining in healthcare and are intended to help address key base data, clinical and technical aspects.

### Appendix A.1. Base Data Aspects

**Table A1:** Aspects that describe the basic characteristics of the data.

Aspect	Description / Example
Data Source	e.g., Administrative system, Clinical support system, Medical devices
Descriptive Statistics	Statistics of the base data; e.g., the number of cases and patients
Timeframe	The period during which the underlying data was collected
Geographical Area	Country or region where the data was collected

### Appendix A.2. Clinical Aspects

**Table A2:** Clinical aspects of the mined healthcare process.

Aspect	Coding Scheme	Listing / Example
Process Type	-	Organizational or medical treatment process; following the definition of Lenz and Reichert [46]
Encounter Type	-	Elective or non-elective care
Encounter Environment	SNOMED CT	see Table 4; e.g., Inpatient (440654001)
Clinical Specialty	SNOMED CT	see Table 5; e.g., Dentistry (722163006)
Diagnosis	ICD 10	e.g., **J10.0** Influenza with pneumonia, seasonal influenza virus identified
Investigations/Procedures	-	e.g., Complete blood count, X-ray imaging, Colonoscopy, Appendectomy

### Appendix A.3. Technical Aspects

**Table A3:** Technical aspects about the process mining techniques.

Aspect	Listing / Example
Type	Discovery, Conformance or Enhancement
Perspective	Control-flow, Organizational, Case, Time
Tools (Version)	e.g., ProM 6.9 or Disco 2.2.1
Implementation Strategy	Direct, Semi-Automated, Integrated Suite
Analysis Strategy	Basic, New implementation, Extended Analysis
Methodology	e.g., L* life-cycle model
Techniques/Algorithms	e.g., Genetic Process Mining, Inductive Mining
